# An Enhanced Circularly Polarized Textile Antenna Using a Metasurface and Slot-Patterned Ground for Off-Body Communications

**DOI:** 10.3390/mi16070799

**Published:** 2025-07-09

**Authors:** Yong-Deok Kim, Tu Tuan Le, Tae-Yeoul Yun

**Affiliations:** Department of Electronic Engineering, Hanyang University, Seoul 04763, Republic of Korea

**Keywords:** CP antenna, LP-to-CP converter, metasurface, off-body, SAR, slot-patterned ground, wearable antenna

## Abstract

This paper presents an enhanced circularly polarized (CP) all-textile antenna using a metasurface (MS) and slot-patterned ground (SPG) for 5.8 GHz industry, scientific, and medical (ISM)-band applications in off-body communications. The 3 × 3 MS, capable of converting the incident wave into an orthogonal direction with equal magnitude and a 90° phase difference, converts the linearly polarized (LP) wave, radiated from the fundamental radiator with a corner-truncated slot square-patch configuration, into being CP. The SPG, consisting of periodic slots with two different sizes of corner-truncated slots, redistributes the surface current on the ground plane, enhancing the axial ratio bandwidth (ARBW) of the proposed antenna. The novel combination of MS and SPG not only enables the generation and enhancement of CP characteristics but also significantly improves the impedance bandwidth (IBW), gain, and radiation efficiency by introducing additional surface wave resonances. The proposed antenna is composed of a conductive textile and a felt substrate, offering comfort and flexibility for applications where the antenna is placed in close proximity to the human body. The proposed antenna is simulated under bending in various directions, showing exceptionally similar characteristics to a flat condition. The proposed antenna is fabricated and is then verified by measurements in both free space and a human body environment. The measured IBW is 36.3%, while the ARBW is 18%. The measured gain and radiation efficiency are 6.39 dBic and 64.7%, respectively. The specific absorption rate (SAR) is simulated, and the results satisfy both US and EU safety standards.

## 1. Introduction

Numerous wearable devices have been widely used in industrial, scientific, and medical (ISM)-band applications. In these applications, the communication link between wearable devices and a central monitor should be maintained stably and reliably. A wearable antenna is an important essential component of that function and needs to be carefully designed because it works in proximity to the human body.

Circularly polarized (CP) antennas, with their distinct advantages over linearly polarized (LP) antennas including mitigating the multi-path propagation effect and reducing polarization mismatch, are frequently adopted in modern communication systems [1]. These wearable CP antennas have various shapes such as slot antennas [2,3,4], crossed-dipole antennas [5,6,7,8], and spiral antennas [9]. A slot on the patch generates CP waves by adjusting its length, width, and position. However, the adjustment affects the antenna parameters such as resonant frequency and impedance bandwidth. Eventually, the antenna configuration becomes complex and minimizes side effects. Crossed-dipole and spiral antennas have large profiles. Recently, a metasurface (MS) has been adopted in antenna design to enhance antenna performance including the impedance bandwidth (IBW), radiation efficiency, and axial ratio bandwidth (ARBW) [10,11,12]. In addition, a MS can be used as a polarization converter to convert LP to CP waves [13,14,15]. In this method, the MS reflects the incident LP wave in an orthogonal direction with a 90° phase shift. The reflected wave is combined with a 0° phase incident wave and is turned into a CP wave. As a result, the incident LP wave can be converted into a CP wave. The polarization conversion MS has also been studied for wearable applications [16,17]. However, there are no studies on deformation, which is one of the essential characteristics of wearable applications. Moreover, their antenna size is not compact enough to ignore deformation occasions. The authors of [18,19] conducted bending simulations along the x- and y-directions, and their antennas exhibit a comparatively more compact size than those in [16,17]. However, their configurations feature an asymmetrical design along the oblique direction of y = x and y = −x, which may lead to the reduced reliability under physical deformation that frequently occurs in wearable applications.

Modified ground plane structures such as slot-patterned ground (SPG) and defected ground structure (DGS) have been widely employed in microstrip patch antenna design to improve electromagnetic performance, including impedance bandwidth (IBW), axial ratio bandwidth (ARBW), radiation efficiency, and gain. These ground modifications influence the surface current distribution and contribute to the excitation of multiple resonant modes or polarization control. For instance, multiple slots in the SPG were used to achieve wideband behavior in a compact design [18], while a defected ground plane combined with a slotted patch enabled a compact omnidirectional antenna with enhanced bandwidth [19]. PIN diode-integrated slots allowed for reconfigurable circular polarization with an improved bandwidth and axial ratio [20]. Additionally, asymmetrical slots [21] and modified ground structures with parasitic patches [22] contributed to ARBW extension and gain improvement. These designs have primarily been demonstrated under rigid or planar conditions, and their suitability for physically dynamic scenarios remains uncertain. In other studies, the bending performance of antennas employing DGS [23,24] and a slotted ground [25] has been investigated. However, the bending scenarios reported by the authors of [23,24] were limited to a single direction. Although the authors of [25] conducted multi-directional bending evaluations and preserved CP performance, the antenna exhibited significant directional sensitivity, as evidenced by variations in the axial ratio between x-axis and y-axis bending. This behavior is attributed to its asymmetric structure as is the case in the work reported in [23,24], which causes the uneven distortion of current paths under deformation.

In this study, an all-textile CP antenna is proposed. This study proposes a polarization conversion MS and novel combination with a symmetric SPG composed of non-uniform slots, primarily enhancing ARBW, while also improving IBW, gain, and radiation efficiency. The antenna is fabricated with textiles for full flexibility, which is one of the important characteristics of wearable applications. Initially, the corner-truncated slot square-patch antenna is designed to radiate LP waves as a fundamental radiator. Later, a 3 × 3 symmetrical square-ring MS is placed above the fundamental patch radiator. Then, the MS converts LP waves into CP waves. In addition, the MS enhances the IBW, broadside gain, and radiation efficiency. Later, a periodic slot is applied on the ground plane and redistributes the surface current of the ground plane. This addresses the extra enhancement of antenna performances compared to those of the MS-loaded version. The proposed antenna is simulated over flat and bending conditions, showing stable performances under various physical deformations along the x-, y-, y = x, and y = −x directions. The simulated specific absorption rate (SAR) satisfies US and European standards. The proposed antenna is fabricated with a high accuracy using laser cutting and is measured in free space on a pork-as-a-human phantom. This work contributes to off-body communications with a novel antenna design that achieves a wideband CP performance, remains stable under bending, and satisfies SAR standards.

The rest of this paper is organized as follows. The antenna structure, working mechanism, bending evaluation, parametric study, and SAR simulation are described in Section 2. The measurement results are discussed in Section 3. Finally, conclusions are provided in Section 4.

## 2. Antenna Design

### 2.1. Antenna Structure

**Figure 1:**
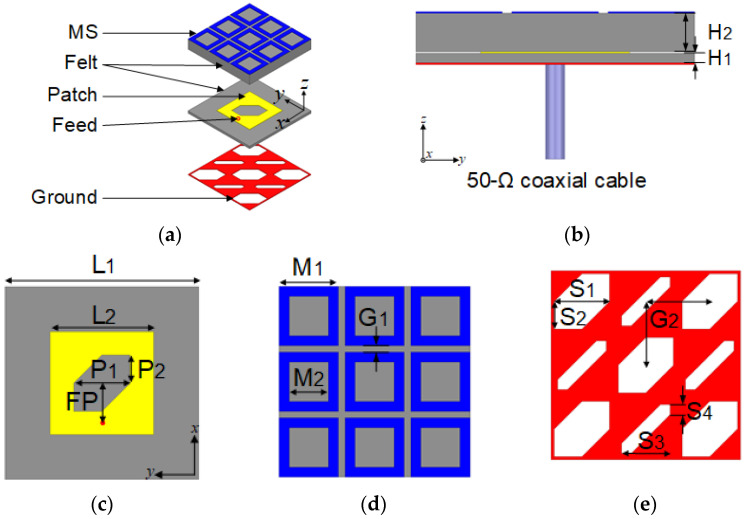
Geometry of the proposed antenna. (**a**) Perspective view; (**b**) side view; (**c**) top view of the patch; (**d**) top view of the MS; (**e**) top view of the slot-patterned ground.

Figure 1 shows the proposed antenna structure, which is composed of felt substrates and conductive textiles. The felt substrate has a dielectric constant of 1.4 and a loss tangent of 0.044. The conductive textile has a conductivity of 118,000 S/m and a thickness of 0.17 mm. The complete antenna has two layers—a radiator at the bottom and an MS on the top. The radiator has a corner-truncated slot square-patch and an SPG at the top and bottom, respectively, on a 1-mm-thick felt substrate. The radiator is fed using a 50 Ω coaxial cable. The MS has symmetrical 3 × 3 arrays of a square-ring conductive textile unit-cell on a 4-mm-thick felt substrate. The overall dimensions of the proposed antenna are 0.56 × 0.56 × 0.09 $\lambda_{0}^{3}$ (29 × 29 × 5 mm^3^), where $\lambda_{0}$ is the wavelength at the lowest frequency over the IBW. The optimized dimensions of the proposed antenna are H_1_ = 1, H_2_ = 4, L_1_ = 29, L_2_ = 15.5, P_1_ = 8.5, P_2_ = 4.25, S_1_ = 8.5, S_2_ = 4.25, S_3_ = 7.5, S_4_ = 1.5, M_1_ = 9, M_2_ = 6, G_1_ = 1, G_2_ = 9.8, and FP = 6 (unit: mm).

### 2.2. Working Mechanism

#### 2.2.1. Metasurface

**Figure 2:**
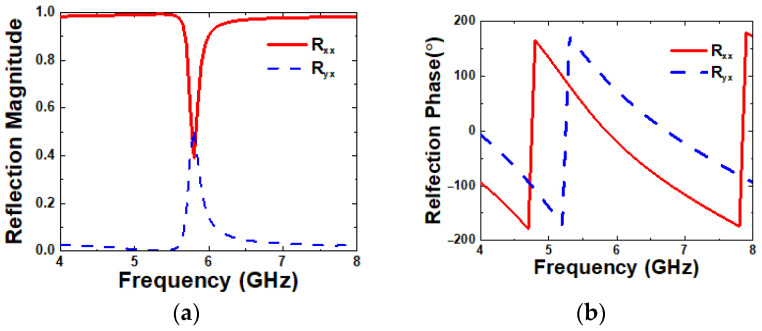
Reflection coefficient of the unit-cell in an x-polarized normal incident wave. (**a**) Magnitude and (**b**) phase.

The MS consists of unit-cell arrays after evaluating the reflection characteristics of a single unit-cell. In this study, a square-ring unit-cell is adopted due to the ease of adjusting the working frequency of the MS by simply modifying the external length, M_1_, and internal length, M_2_. The unit-cell is analyzed using a Floquet-port model for its reflection characteristics [26]. In the unit-cell analysis, R_xx_ and R_yx_ represent the reflection coefficients from an x-polarized incident wave to an x- and y-polarized reflect wave, respectively, which are plotted in Figure 2. As shown in Figure 2a, the y-polarized reflection (R_yx_) is excited, while the x-polarized reflection (R_xx_) is suppressed at 5.8 GHz, which means that an x-polarized incident wave is more highly reflected as a y-polarized wave than an x-polarized wave. The phase of the reflection coefficient is shown in Figure 2b. The reflection phase, R_yx_, at 5.8 GHz is almost equal to 90°, which means that the incident wave lags 90° behind the y-polarized reflected wave. Due to the 90° phase difference, which is 90° polarization rotation, right-handed circular polarization (RHCP) can be achieved by combining the incident wave with the reflected wave.

Since the square-ring unit-cell is analyzed and confirmed to function well, it is arrayed as 3 × 3, consisting of the MS loaded on the square-patch, as shown in Figure 1. The square-patch radiates an x-polarized LP wave onto the MS. Then, the MS polarizes the incident wave to the y-direction with a 90° phase difference. The y-polarized reflected wave is transmitted through the MS and combined with an x-polarized incident wave. The combined wave introduces a CP wave.

**Figure 3:**
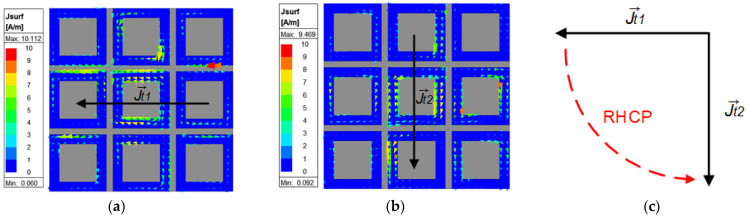
Surface current distributions on MS at 5.8 GHz. (**a**) t1 = 0, (**b**) t2 = T/4, and (**c**) vector rotation.

The simulated surface current distributions on the MS at 5.8 GHz are shown in Figure 3, where T is the time period, $\vec{J_{t1}}$ at t = 0, and $\vec{J_{t2}}$ at t = T/4. As shown in Figure 3c, the curved dashed red arrow traces the peaks of $\vec{J_{t1}}$ and $\vec{J_{t2}}$. This trace indicates a counter-clockwise rotation of the surface current vector, confirming that the MS-loaded patch antenna generates a right-hand circularly polarized (RHCP) wave.

**Figure 4:**
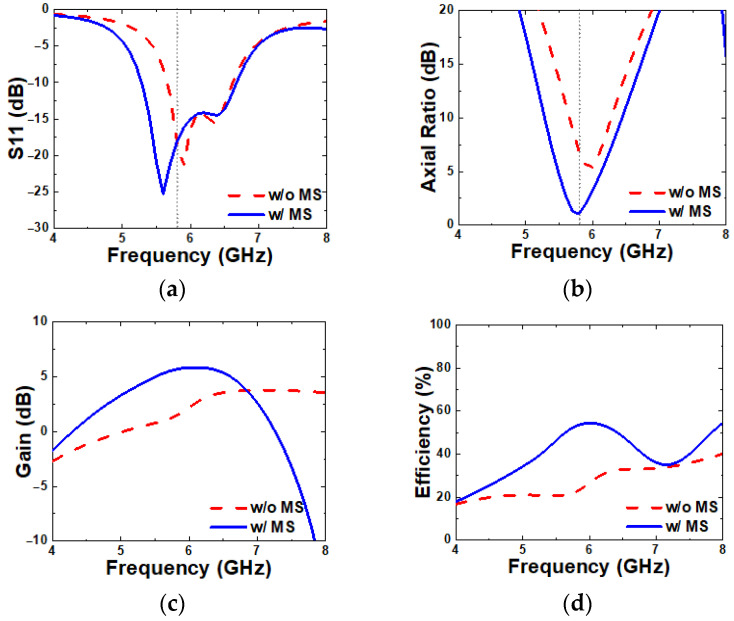
Simulated (**a**) S11, (**b**) ARBW, (**c**) gain, and (**d**) radiation efficiency without and with an MS.

Comparisons of the simulated S11, axial ratio bandwidth, broadside gain, and radiation efficiency between the patch antenna with and without the MS are presented in Figure 4 to evaluate the effect of the MS. The dotted lines are to indicate the target frequency of 5.8 GHz. The patch antenna, which includes a full ground plane, shows a −10 dB IBW of 15.6% (5.65–6.61 GHz) in Figure 4a. When the patch antenna is integrated with the MS, it results in an IBW of 23.1% (5.27–6.64 GHz). The simulated axial ratio without the MS remained around 7 dB, indicating linear polarization, while the MS-loaded patch antenna achieves circular polarization with an ARBW of 6.8% (5.61–6.01 GHz), as shown in Figure 4b. In addition, the gain and radiation efficiency at 5.8 GHz for the patch with the MS are improved to 5.65 dB and 55%, respectively, as shown in Figure 4c,d. These results confirm that the use of a periodic patterned MS on the substrate significantly enhances the performance of the patch antenna.

#### 2.2.2. Slot-Patterned Ground

**Figure 5:**
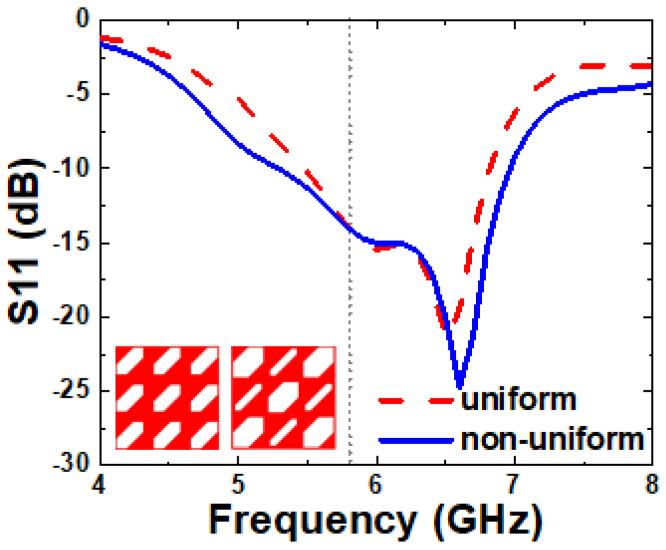
Simulated S11 of uniform and non-uniform slots.

The MS-loaded patch antenna initially exhibits a relatively narrow ARBW of 6.8%, which limits its polarization performance. To address this, an SPG is introduced beneath the radiating element to enhance the ARBW. The SPG is initially designed with an equal size or uniform truncated slot, inspired by the work reported in [27]. Later, to improve the IBW, the proposed SPG consists of two different sizes or non-uniform slots. The smaller slots shift to the higher frequency resonance (6.81 to 6.96 GHz), while the larger slots shift to the lower frequency (5.48 to 5.29 GHz), as shown in Figure 5. The distance between slots (G2) is optimized through simulations to control the capacitive coupling between them and is selected as 9.8 mm.

**Figure 6:**
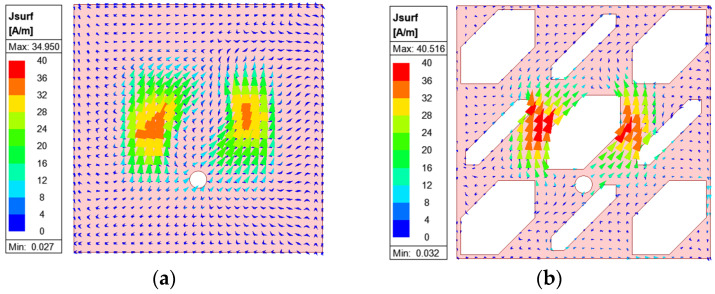
Simulated surface current distributions on (**a**) SG and (**b**) SPG at 6.1 GHz.

These slots are strategically arranged to perturb the surface currents flowing on the solid ground (SG), thereby generating new current paths in the vicinity of the added slots. As shown in Figure 6, the modified current distribution on the SPG of non-uniform slots reveals a strong concentration of surface currents around the central slot at 6.1 GHz. This also shows noticeable strength along the x-direction, which will increase CP performance and ARBW, as mentioned in the discussion surrounding the MS working mechanism and as demonstrated below.

**Figure 7:**
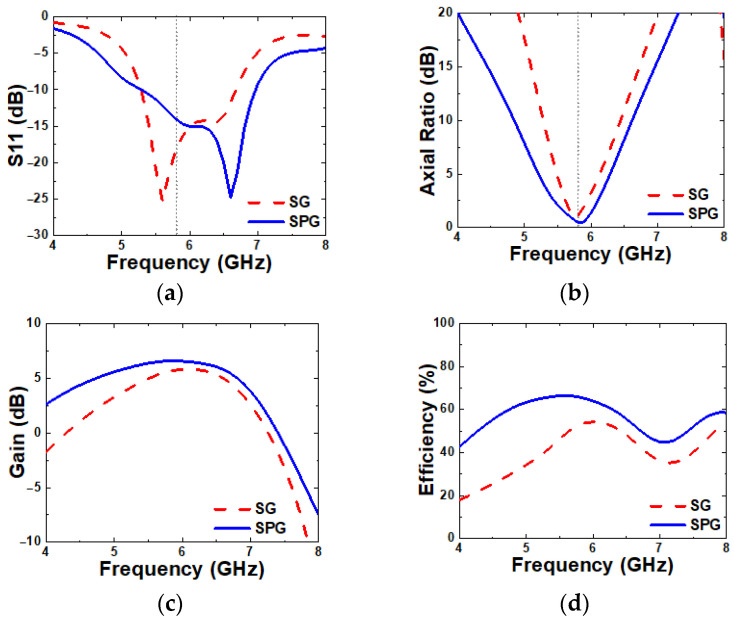
Simulated (**a**) S11, (**b**) axial ratio, (**c**) gain, and (**d**) radiation efficiency of SG and SPG.

Figure 7 compares the simulated S11, ARBW, gain, and radiation efficiency between the proposed antenna with SG and SPG. The proposed antenna with SPG achieves a wider IBW of 27.26% (5.29–6.96 GHz), compared to 23.9% for the SG antenna. The ARBW is also improved significantly with the proposed SPG, reaching 13.07% (5.39–6.14 GHz), whereas the SG antenna achieved only 6.8%. At 5.8 GHz, the proposed SPG antenna shows an increase in gain from 5.65 to 6.59 dB and shows an increase in radiation efficiency from 55% to 65%. These results demonstrate that the introduction of SPG effectively enhances the overall antenna performance.

### 2.3. Bending Evaluation

**Figure 8:**
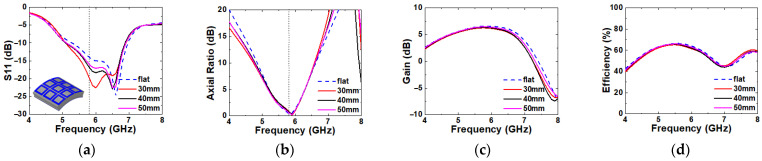

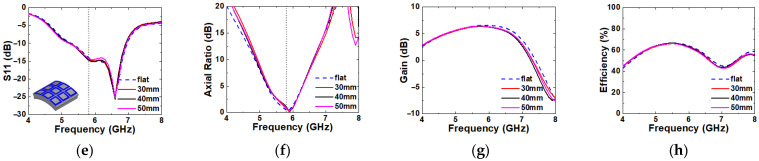
Bending simulation of x- (**a**–**d**) and y-axis (**e**–**h**) directions. (**a**,**e**) S11, (**b**,**f**) axial ratio, (**c**,**g**) gain, and (**d**,**h**) radiation efficiency.

**Figure 9:**
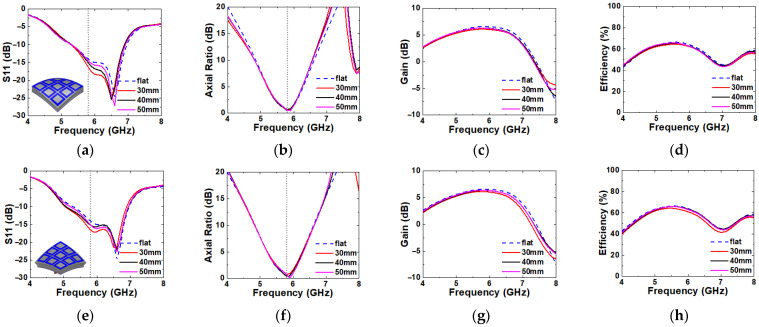
Bending simulation of y = x (**a**–**d**) and y = −x directions (**e**–**h**). (**a**,**e**) S11, (**b**,**f**) axial ratio, (**c**,**g**) gain, and (**d**,**h**) radiation efficiency.

Since the wearable antenna is frequently exposed under multidirectional mechanical stress by human activity, antenna bending simulation is required for various directions of deformation to prove its stable performance. In order to verify the antenna performance in a deformation structure, the IBW, ARBW, gain, and radiation efficiency are simulated over imaginary cylinders with radii of 30, 40, and 50 mm to replicate the size of the human arm, as shown in Figure 8. Since the proposed antenna has a symmetric structure along the x- and y-directions, the simulation results in Figure 8 represent its performance when it is deformed along y-direction only. However, the proposed antenna has an asymmetric structure in the oblique directions along y = x and y = −x. Therefore, additional bending simulations are performed for the two oblique directions, and the results are plotted in Figure 9. In this simulation, all of the antenna elements bend in equal curvature by each radius. A very minor effect is observed for all bending radii and the directions for all of the simulated parameters. Therefore, it is verified that the proposed antenna can work properly in the deformation structure.

### 2.4. Parametric Study

To further analyze the impact of key parameters on antenna performance, parametric studies are conducted. In each case, one parameter is varied while all other parameters are held constant at their optimized values.

**Figure 10:**
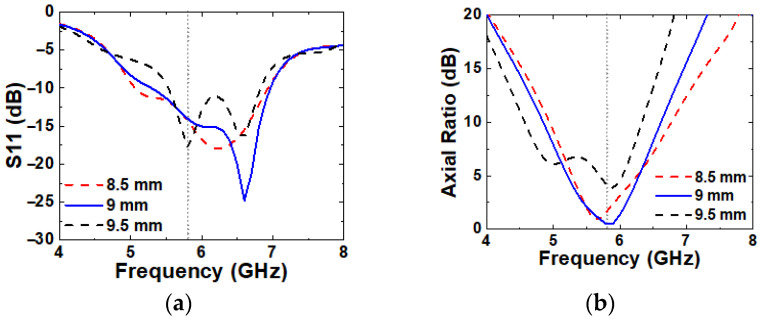
Simulated (**a**) S11 and (**b**) axial ratio with varying M1.

The influence of M1 on antenna performances is shown in Figure 10. As M1 increases, the unit-cells become closer, which increases mutual coupling between unit-cells and disturbs the resonance behavior. As a result, narrower IBW and loss of CP characteristics are observed. Among the tested values, the best performance is achieved at M1 = 9 mm.

**Figure 11:**
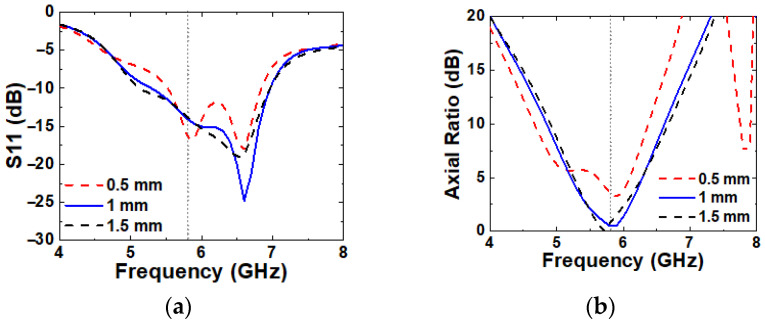
Simulated (**a**) S11 and (**b**) axial ratio with varying G1.

Similarly, the smaller G1 leads to a closer spacing between unit-cells, and as is observed with the effect of a larger M1, a narrower IBW and the disappearance of CP are observed, as shown in Figure 11. The best performance is achieved at G1 = 1 mm.

**Figure 12:**
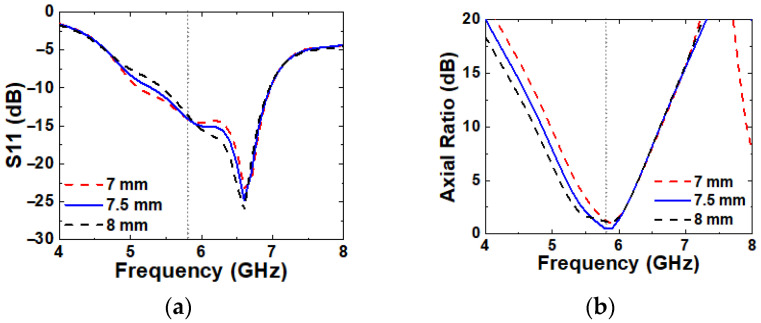
Simulated (**a**) S11 and (**b**) axial ratio with varying S3.

The variation in S3 shows minor differences in S11 and axial ratio curves, as shown in Figure 12. A larger S3 results in a narrower IBW and a wider ARBW, while a smaller S3 acts in the opposite way. The best performance is achieved at S3 = 7.5 mm. The variation alters the current distribution on the SPG and causes minor effects on S11 and axial ratio.

**Figure 13:**
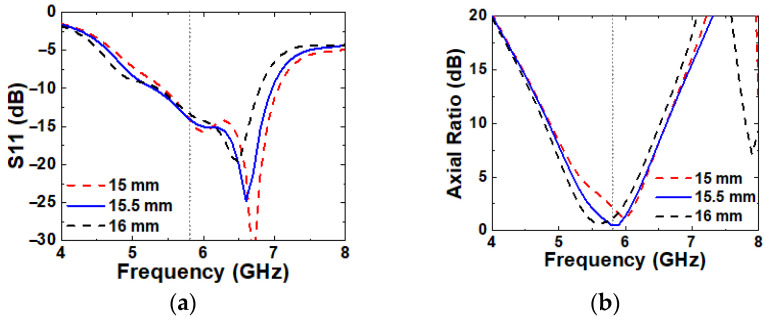
Simulated (**a**) S11 and (**b**) axial ratio with varying L2.

The influence of L2 on antenna performances is shown in Figure 13. A larger L2 shifts both S11 and axial ratio curves to lower frequencies due to the longer current path, while a smaller L2 shifts them to higher frequencies due to the shorter path. The best performance is achieved at L2 = 15.5 mm.

**Figure 14:**
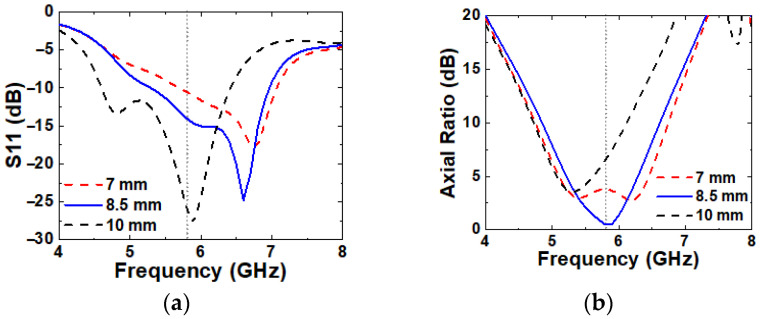
Simulated (**a**) S11 and (**b**) axial ratio with varying P1.

A larger P1 causes a noticeable shift in both S11 and axial ratio to lower frequencies, as shown in Figure 14. A smaller P1 results in a narrower IBW and the loss of CP characteristics, likely due to modified current distribution.

### 2.5. SAR Simulation

**Figure 15:**
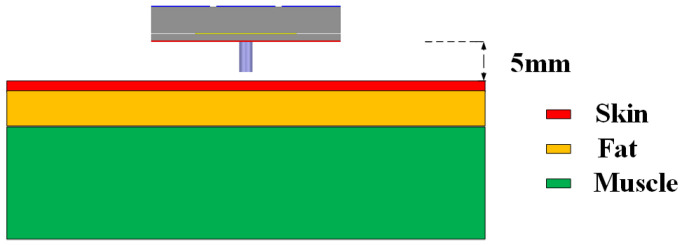
Arrangement of the antenna and human tissue in the SAR simulation.

**Figure 16:**
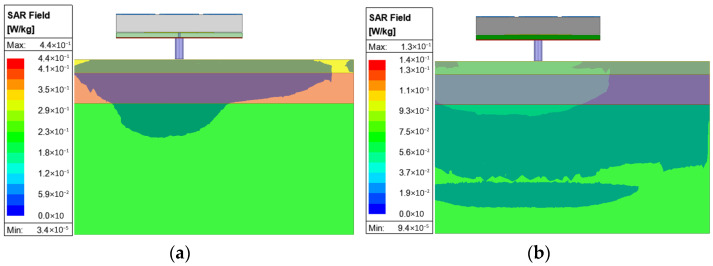
Simulated SARs at 5.8 GHz for (**a**) the US standard and (**b**) the European standard.

In wearable applications, the SAR is a very important parameter that ensures the safety of the wearer. The US standard is a limit of 1.6 W/kg for a 1 g average mass, while the European limit is 2 W/kg for a 10 g average mass. The simulation is performed with a human tissue model, as shown in Figure 15. The permittivity, conductivity, and mass density of skin, fat, and muscle were 35.11, 3.72 S/m, and 1100 kg/m^3^; 4.85, 0.29 S/m, and 910 kg/m^3^; and 48.84, 4.96 S/m, and 1041 kg/m^3^, respectively. The tissue model had dimensions of 80 × 80 × 40 mm^3^. The thicknesses of skin, fat, and muscle were 3, 7, and 30 mm, respectively. The 5 mm gap between the antenna and human tissue accounts for the thickness of the wearer’s clothes. An input power of 100 mW is chosen for the simulation, in which the simulated maximum SARs at 5.8 GHz are 0.44 W/kg for the US standard and 0.13 W/kg for the European standard, as shown in Figure 16. For both standards, the simulated SARs were significantly lower than the limits.

## 3. Measurement Results and Discussion

**Figure 17:**
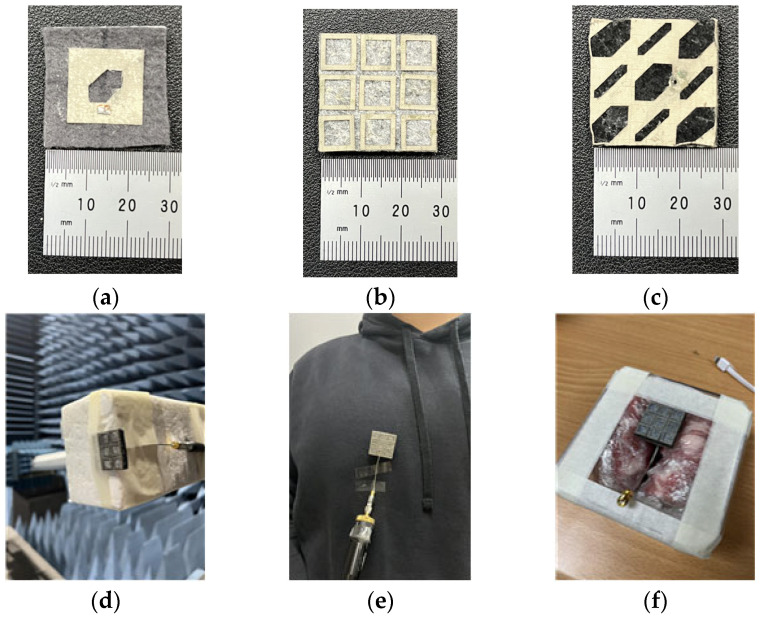
Fabricated proposed antenna and measurement scenes. (**a**) Patch radiator, (**b**) MS, (**c**) SPG, (**d**) in free space, (**e**) on chest, and (**f**) on pork phantom.

To validate the simulated results and the suitability of the proposed antenna for wearable applications, the antenna is fabricated and measured in free space on a human’s body parts. In addition, the antenna is also measured on pork in an anechoic chamber, which is used as a replacement for a human phantom. Figure 17 presents the fabricated antenna and measurement setup of assembled antenna. Figure 17a–c display the radiating patch, MS, and SPG, respectively. Figure 17d–f illustrate antenna measurement scenarios in free space, on a human chest, and on the phantom, respectively.

**Figure 18:**
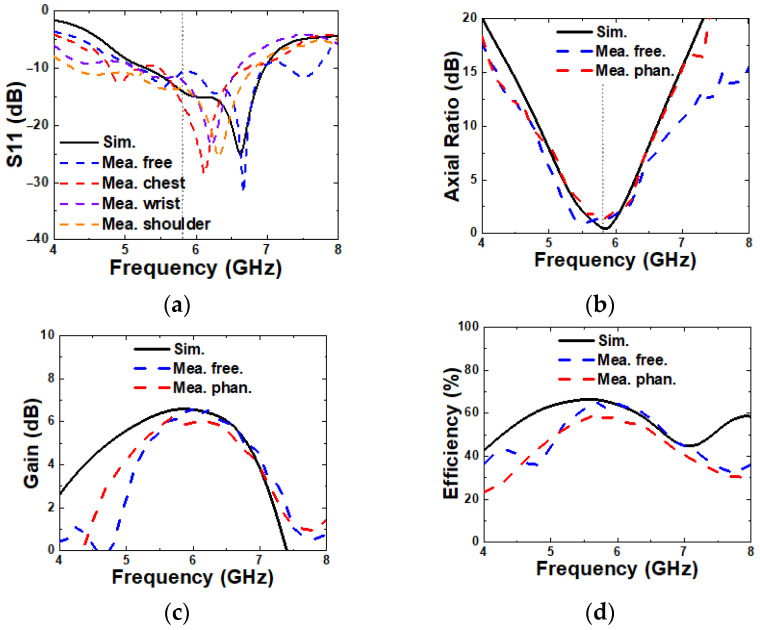
Simulated and measured (**a**) S11, (**b**) axial ratio, (**c**) gain and radiation efficiency, and (**d**) radiation pattern.

In Figure 18a, the measured S11 values in free space, as well as on chest, wrist, and shoulder, are shown. The measurement in free space, at 28.9% (5.18–6.93 GHz), shows good agreement with the simulation, while the measurements on the human chest, wrist, and shoulder with different curvatures exhibit a slight downshift in frequency due to the bending effect. Nonetheless, the overall impedance bandwidth remains comparable, indicating the robustness and stability of the proposed antenna under different operating conditions. A discrepancy in S11 magnitude between simulation and measurement is observed, which is primarily attributed to fabrication tolerances and variations in the experimental setup.

In Figure 18b, the measured 3 dB axial ratio bandwidths (ARBWs) in free space and on the phantom are 18% (5.21–6.24 GHz) and 13.3% (5.46–6.24 GHz), respectively. These results exhibit good agreement with the simulated ARBW, confirming a consistent circular polarization performance.

In Figure 18c and Figure 18d, the measured broadside gain and radiation efficiency at 5.8 GHz are 6.39 dBic and 64.7% in free space, and 5.9 dBic and 57.7% on the phantom, respectively. While the measurements in free space closely match the simulations, the results obtained on the phantom show moderate degradation. This reduction is likely due to the lossy and high-permittivity characteristics of the phantom material.

**Figure 19:**
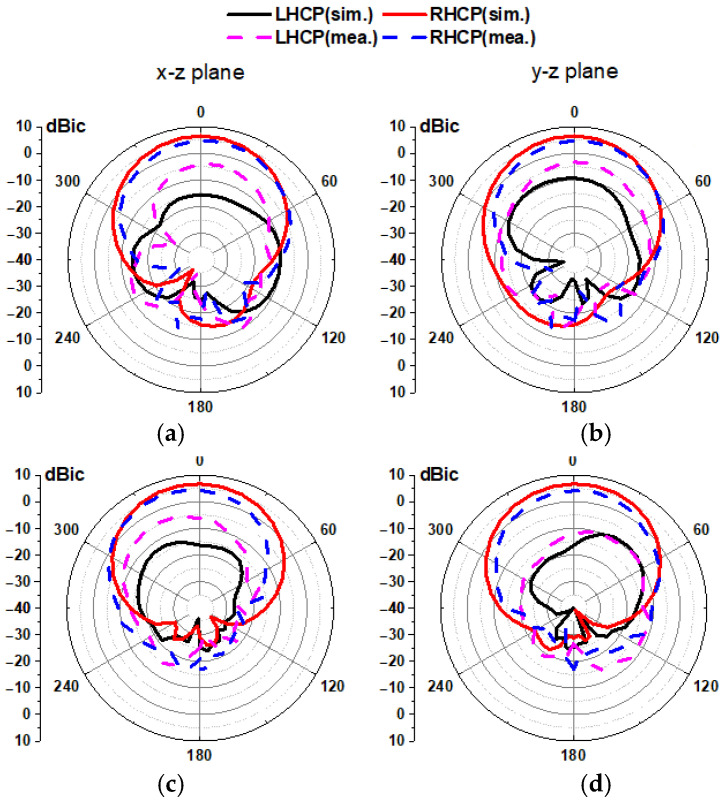
Simulated and measured radiation pattern at 5.8 GHz. (**a**,**b**) In free space and (**c**,**d**) on phantom.

In Figure 19, the measured radiation patterns in free space and on the phantom at 5.8 GHz in the x-z and y-z planes are plotted. As a result, the proposed antenna radiates RHCP waves in a broadside direction and shows good agreement with simulated results both in free space and on phantom scenarios. The measured cross-polarization discrimination is 31.2 dB, while the measured front-to-back ratio is 19.2 dB.

The comparison of the proposed antenna performances with those of other wearable CP antennas is shown in Table 1 [28,29,30,31,32]. The proposed antenna has a lower radiation efficiency than others, which likely arises due to its textile material characteristics. However, the proposed antenna achieves a high ARBW and gain, along with an exceptionally wide IBW, while maintaining a competitive size compared to other studies due to the MS and SPG structures. The SPG satisfied the international standard of SAR, even without a solid ground plane. While other studies, despite asymmetric designs, examine only the x- and y-directions, this work analyzes along the x-, y-, x = y, and x = –y directions, showing consistent results with the flat condition.

## 4. Conclusions

A circularly polarized low-SAR all-textile wearable antenna for the 5.8 GHz ISM-band was proposed. The antenna comprises a corner-truncated slot square-patch radiator and a 3 × 3 square-ring unit-cell MS. The polarization converter MS receives LP waves from the radiator, polarizes incident waves orthogonally, and introduces CP waves. The MS effectively enhances the IBW, antenna gain, and radiation efficiency. The novel combination with SPG introduces extra antenna performance enhancement. Moreover, the bending evaluations reveal a small effect on antenna deformation. The SAR simulation demonstrates that the antenna satisfies the SAR limits for both US and European standards. The proposed antenna is validated by measurements in free space and on human phantom environments, showing good agreement. The experimental results indicate that the proposed antenna is a suitable candidate for off-body communications.

## Figures and Tables

**Table 1:** Comparison with other wearable CP antennas.

Ref.	[28]	[29]	[30]	[31]	[32]	This Work
IBW (%)	18.3	11.44	7.6	15.9	14	36.3
ARBW (%)	18.3	18.18	10.3	2.72	7	18
Eff. (%)	90	94.69	83	79	70.8	64.7
Gain (dBic)	6.2	7.6	1.98	5.2	3.5	6.39
SAR (W/kg) *	0.29	0.02	N.A	0.18	N.A	0.44
Flexibility	Semi	Semi	Full	Full	Semi	Full
Bending **	x-/y-	x-	x-/y-	y-	NA	x-/y-/x = y/x = −y
$\mathrm{Size}\;\left(\lambda_{0}^{3}\right)$	0.5 × 0.5 × 0.06	0.62 × 0.62 × 0.007	0.7 × 0.7 × 0.04	0.4 × 0.4 × 0.04	1.8 × 1.8 × 0.22	0.56 × 0.56 × 0.09

* The SAR data of [28,29,31] were recalculated with a 100 mW input power based on the US standard. ** The directions of bending evaluation.

## Data Availability

The original contributions presented in this study are included in the article. Further inquiries can be directed to the corresponding author.
